# Phytoconstituent-Loaded Nanofibrous Meshes as Wound Dressings: A Concise Review

**DOI:** 10.3390/pharmaceutics15041058

**Published:** 2023-03-24

**Authors:** Ameya Sharma, Divya Dheer, Inderbir Singh, Vivek Puri, Pradeep Kumar

**Affiliations:** 1 Chitkara School of Pharmacy, Chitkara University, Baddi 174103, Himachal Pradesh, India; 2 Chemical Biology Unit, Institute of Nano Science and Technology, Knowledge City, Sector 81, Mohali 140306, Punjab, India; 3 Chitkara College of Pharmacy, Chitkara University, Rajpura 140401, Punjab, India; 4 Department of Pharmacy and Pharmacology, School of Therapeutic Sciences, Faculty of Health Sciences, University of the Witwatersrand, Johannesburg 2050, South Africa

**Keywords:** phytoconstituents, nanofibers, wound, wound healing, wound dressings, polysaccharides

## Abstract

In the past, wounds were treated with natural materials, but modern wound dressings include functional elements to expedite the process of healing and to improve skin recovery. Due to their exceptional properties, nanofibrous wound dressings are now the most cutting-edge and desirable option. Similar in structure to the skin’s own extracellular matrix (ECM), these dressings can promote tissue regeneration, wound fluid transportation, and air ductility for cellular proliferation and regeneration owing to their nanostructured fibrous meshes or scaffolds. Many academic search engines and databases, such as Google Scholar, PubMed, and Sciencedirect, were used to conduct a comprehensive evaluation of the literature for the purposes of this investigation. Using the term “nanofibrous meshes” as a keyword, this paper focuses on the importance of phytoconstituents. This review article summarizes the most recent developments and conclusions from studies on bioactive nanofibrous wound dressings infused with medicinal plants. Several wound-healing methods, wound-dressing materials, and wound-healing components derived from medicinal plants were also discussed.

## 1. Introduction

The treatment of wounds has become extremely difficult due to the complexities involved in wound-healing processes and the requirement to deal with paradigms outside of clinical contexts, which has led to the difficulty of wound therapy [1]. Human skin is essential for physiological functions such as fluid balance, thermoregulation, immunological surveillance, self-healing, sensory detection, and so on [2]. The disruption of skin integrity due to injury or infection can induce deficits in skin functioning, all the way up to mortality [3,4]. A wound is a cut, abrasion, or other opening in the skin that has resulted in tissue loss, blood vessel disruption, the loss of bodily fluids, or a lack of oxygen [5,6,7]. In the field of wound-healing physiology, wounds are classified as either acute or chronic. Acute wounds are commonly the result of the sheering, blunting, or hitting action of heavy surfaces [8,9]. Acute wounds can result from explosions, fires, and other accidents, as well as through contact with caustic chemicals or electricity [10]. In fact, most acute wounds completely heal in around 8 to 12 weeks with minimal scarring [11,12]. Some chronic wounds can take up to 12 weeks to heal [13,14]. Ulcers result from prolonged illness, repetitive trauma to the tissues, and delayed treatment [15,16] to these wounds. The presence of anoxia, hypoxia, lesions, cell debris, and systemic barriers all play a role in the development of chronic wounds [17]. Both acute and chronic wounds might be complicated if they include the following characteristics:

a. Substantial erosion of the epithelium, including hair, skin, and related glands;
b. Infection of the wound may potentially result in the loss of integument [18];
c. Underlying bacterial growth is present in wounds; hence, if the host is incapable of inhibiting the development of bacteria, the wound would lead to develop an infection [19].

**Figure 1:**
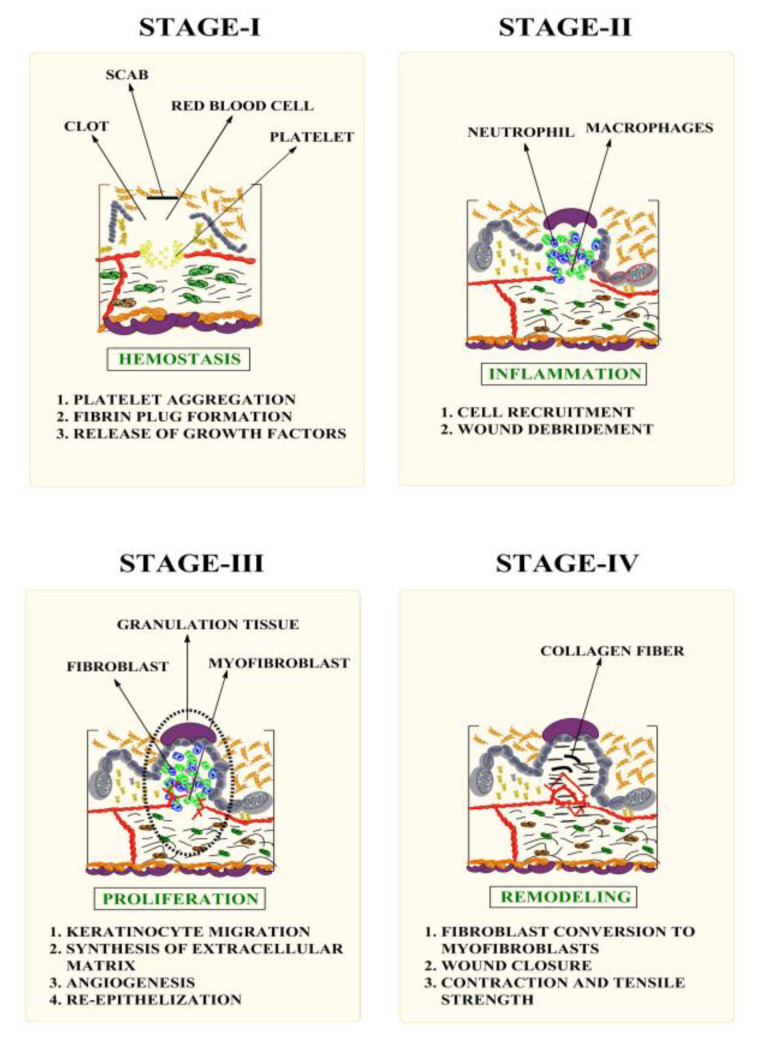
Various stages of the wound-healing mechanism.

Symptoms such as erythema, swelling, pain, and exudate, as well as loss of functionality, are all signs that allude to the prevalence of a local infection [20]. Accelerated epithelialization and overall wound healing have been attributed to a wet environment, where the wound surface behaves as a highly permeable membrane. It is possible to incorporate different effective treatments into the moisture at predetermined concentrations. This gives rise to the possibility of conducting in-depth analyses of any metabolites produced by the wound-healing process, as well as determining the rate of intake and clearance. Wet conditions slow the course of injuries by suppressing the inflammatory response. The fastest healing with the fewest abnormalities and the least amount of scarring is achieved in a wet or moist incubator-like microenvironment. Staphylococcus aureus and anaerobes are the most common causes of chronic wound infections [21]. Adequate quality wound dressing entails maximizing the patient’s systemic local circumstances, as well as fabricating optimal conditions for the healing of wounds [22]. In order to repair and restore damaged tissue, a complex process of wound healing entails many different cellular and biological components and enzyme pathways [23]. First aid is administered at the site of injury and then it is determined how far the wound extends [24]. There are four phases of wound healing: (a) coagulation and hemostasis; (b) inflammation; (c) proliferation; (d) remodeling [25]. Multiple wound-healing stages are shown in Figure 1.

A wound dressing’s primary function, in addition to acting as a protective covering (shielding the wound), is to expedite the healing process [26]. Until the mid-twentieth century, it was assumed that if wounds were kept dry and uncovered, they would heal quickly [27]. However, currently advanced wound dressings (phytoconstituent-loaded dressings) are designed in order to prevent the wound from drying out as well as to aid in healing [28]. An excellent wound dressing ought to be capable of maintaining a warm and moist environment while also providing a variety of exact functions based on the armature winding of wound, the infection, the healing situation, and patient age [29]. Traditional wound-dressing materials are used to prevent infection, but they are now being investigated as platforms for delivering bioactive substances to wound sites [30]. Gauze, cotton, and wool (the traditional wound dressings) did not contribute to the process of healing [31]. These dressings are fabricated to be biologically active, either of their polymeric composition or through the release of bioactive components [32].

Healing and treating wounds are two areas where plants hold enormous potential. In many countries, traditional medicine and folk remedies utilize a wide variety of plants to treat wounds and burns. Tissue damage is repaired and new tissue is grown as a result of these natural compounds. Because of their low risk profile and low cost, phytomedicine is becoming increasingly popular. Scientists have been looking into plants for their possible wound-healing qualities because of the abundance of life-sustaining components they contain [33]. Research in phytopharmaceutical laboratories is currently focused on elucidating the active ingredients and mechanisms of action of a wide range of medicinal plants [34]. The bioactive phytochemical elements of these plants are what give them their therapeutic value, as they have a measurable effect on the human body [35]. Research on developing herbal compounds, incorporated with wound-dressing materials, has increased recently due to health issues with synthetic chemicals being used in medicines and indeed due to the emergence of antibiotic-resistant bacteria [36].This review will explore, as well as explain, the fundamental concepts of the wound-healing process, as well as provide an update on recent developments in phytoconstituent-incorporated nanofibrous wound dressings.

## 2. Fabrication of Nanofibrous Meshes

**Figure 2:**
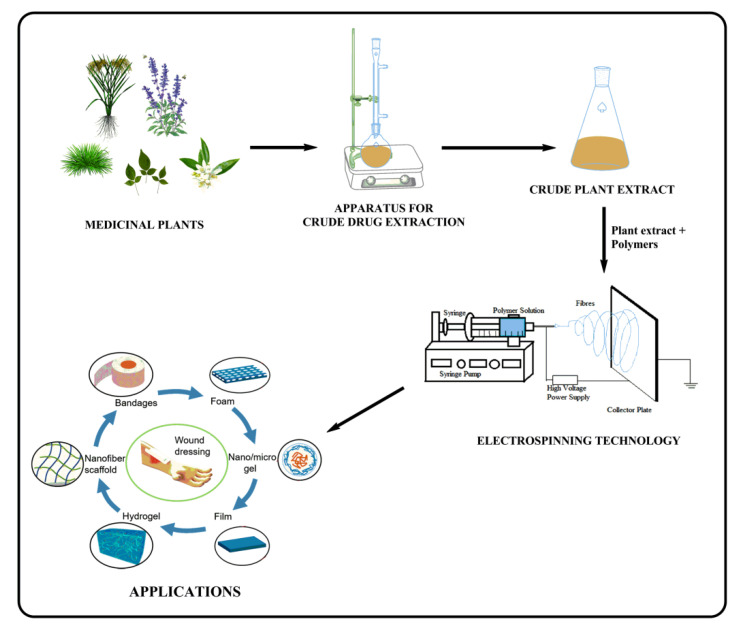
Extraction of phytoconstituents from medicinal plants and fabrication of nanofibrous meshes employing electrospinning and its applications.

Recent advances in nanotechnology suggest that numerous manufacturing procedures are now accessible in order to fabricate scaffolds based on nanofibrous meshes [37]. To generate an appropriate scaffold for the restoration of tissue, each of these approaches relies on the physical, chemical, thermal, and electrostatic interaction of different materials [38]. Electrospinning, self-assembly, as well as phase separation are the three methods that are most commonly utilized in the process of fabricating scaffolds in the fields of tissue engineering and drug delivery [39]. Other methods include framing, drawing, extraction, vapor phase polymerization, reaction kinetics controlled solution synthesis, and chemical oxidative polymerization [40]. These techniques are employed far less frequently than the others; electrospinning, on the other hand, is considered to be the technique that has been employed the most frequently due to its simple operation and excellent reproducibility [41,42]. Various methodologies may be employed to integrate the polymers in electrospun nanofibrous meshes, as shown in Figure 2. When polymers are electrospun together while being combined in a single syringe, the outcome is the formation of fibers having at least two components scattered at random within each fiber [43]. Multi-jet electrospinning produces meshes made of two or more different types of nanofibers by filling each polymer separately in a syringe and electrospinning it separately [44]. These nanofibers may be electrospun simultaneously and randomly distributed (i.e., mixing electrospinning) or arranged into different layers (namely, multilayering electrospinning) [45]. Two separate solutions injected into the outer and inner chambers of a co-axial syringe are spun to construct hybrid nanofibers with a core–shell structure in co-axial electrospinning [46]. Finally, a secondary coating of additional polymers or bioactive materials can be applied to an electrospun polymer [47]. The section will not cover the full details of the various methods as they have been reviewed by several leading reviews.

## 3. Phytoconstituent-Loaded Nanofibrous Meshes

Scientists are increasingly using medicinal plants, herbal extracts, and natural bioactive components instead of synthetic medical plants to produce safer and more accessible treatments for many ailments and to preserve ancient herbal medicine expertise [48]. Herbal extracts, mostly from plants, include bioactive compounds that target biological molecular targets involved in pathophysiological changes of many diseases [49,50]. Herbal extracts are examined for their antibacterial, antiviral, anti-inflammatory, and anticancer properties [51]. The formulation method of phytoconstituent-loaded nanofibrous meshes is performed using the electrospinning method and hence has proven to be a potential nanocarrier system for wound healing, as shown in Table 1 [52]. 

### 3.1. Curcumin-Loaded Platforms

**Figure 3:**
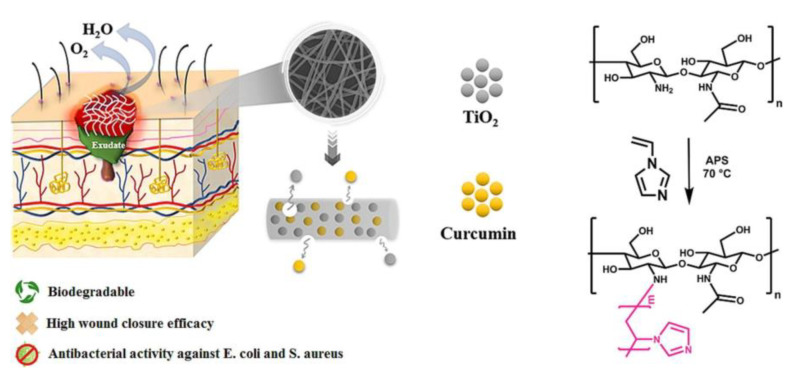
Electrospun Poly(vinyl alcohol)/Chitosan-g-Poly (N-vinyl imidazole) wound dressing containing titanium dioxide/curcumin showing faster wound healing through better drug release (reprinted with permission [55] from Elsevier).

To formulate nanofibrous meshes (membrane) as therapeutic wound dressings, researchers have formulated curcumin-loaded poly L-lactic acid blends by the rotary jet spinning technique and have studied the impact of curcumin in wound-healing applications. They claimed that curcumin-loaded meshes showed compatibility (cellular) with fibroblasts and thus good replacement for conventional chemical-based wound dressings. Wound dressings that provide antioxidants and antimicrobial are desirable for treating infected wounds [53]. In another study, a biopolymer such as gelatin with varying curcumin concentrations was utilized and electrospun and led to the fabrication of a nanofibrous membrane. The formulated membranes demonstrated integrated morphology, efficient water absorption, excellent mechanical properties, and high curcumin dissolution. The average diameters of the PCL/Gel-C0B1, PCL/Gel-C1B1, PCL/Gel-C3B1, and PCL/Gel-C5B1 nanofibers were 711.83, 665.96, 602.49, and 587.25 nm, respectively. With the addition of curcumin, the water contact angle values of the PCL/Gel-C1B1, PCL/ Gel-C3B1, and PCL/Gel-C5B1 nanofiber membranes gradually increased to 88.56 ± 2.51, 92.47 ± 1.09, and 96.71 ± 1.71°, respectively. This increased hydrophobicity was attributed to the highly water-insoluble nature of curcumin. The water vapor transmission rates of the PCL/Gel-C1B1, PCL/Gel-C3B1, and PCL/Gel-C5B1 nanofiber membranes were 0.23, 0.23, 0.24, and 0.25 g/(cm^2^ × 24 h), which is higher than 0.23 g/(cm^2^ × 24 h). The curcumin release rates of the PCL/Gel-C1B1, PCL/Gel-C3B1, and PCL/Gel-C5B1 nanofiber membranes reached 66.02, 80.74, and 74.19%, respectively, which were related to the high specific surface areas of the nanofibers. Curcumin and borneol combat Staphylococcus aureus and dissipate free radicals. The L929 cell line validated the membranes’ biocompatibility. Gelatin-based nanofiber membranes containing curcumin and borneol are potential wound dressings. Biopolymers, as well as environmentally sustainable production procedures, make this process appropriate for commercial composite membrane production [54]. Curcumin has indeed proven to be an antibacterial anti-microbial agent. In another study, researchers have electrospun a Poly(vinyl alcohol)/Chitosan-g-Poly (N-vinyl imidazole) (PVA/CS-g-PNVIM) wound dressing incorporating titanium dioxide/curcumin (CUR) that was developed as a novel wound-healing system with multifunctional features comprising wound closure, drug release, and antimicrobial activities. The nanofiber dressing was mechanically and hydrolytically stable. CS-g-PNVIM-based nanofibers inhibited both Gram +ve and −ve bacteria (Escherichia coli and Staphylococcus aureus) growth by 90% in 1 h without affecting normal fibroblast cells. Animal research demonstrated that CS-g-PNVIM-based nanofibers actually accelerate wound healing as well as tissue regeneration by 14 days. CS-g-PNVIM-based nanofibers are intriguing for compatible antibacterial dressings with appropriate exudate absorption, as shown in Figure 3 [55].

**Figure 4:**
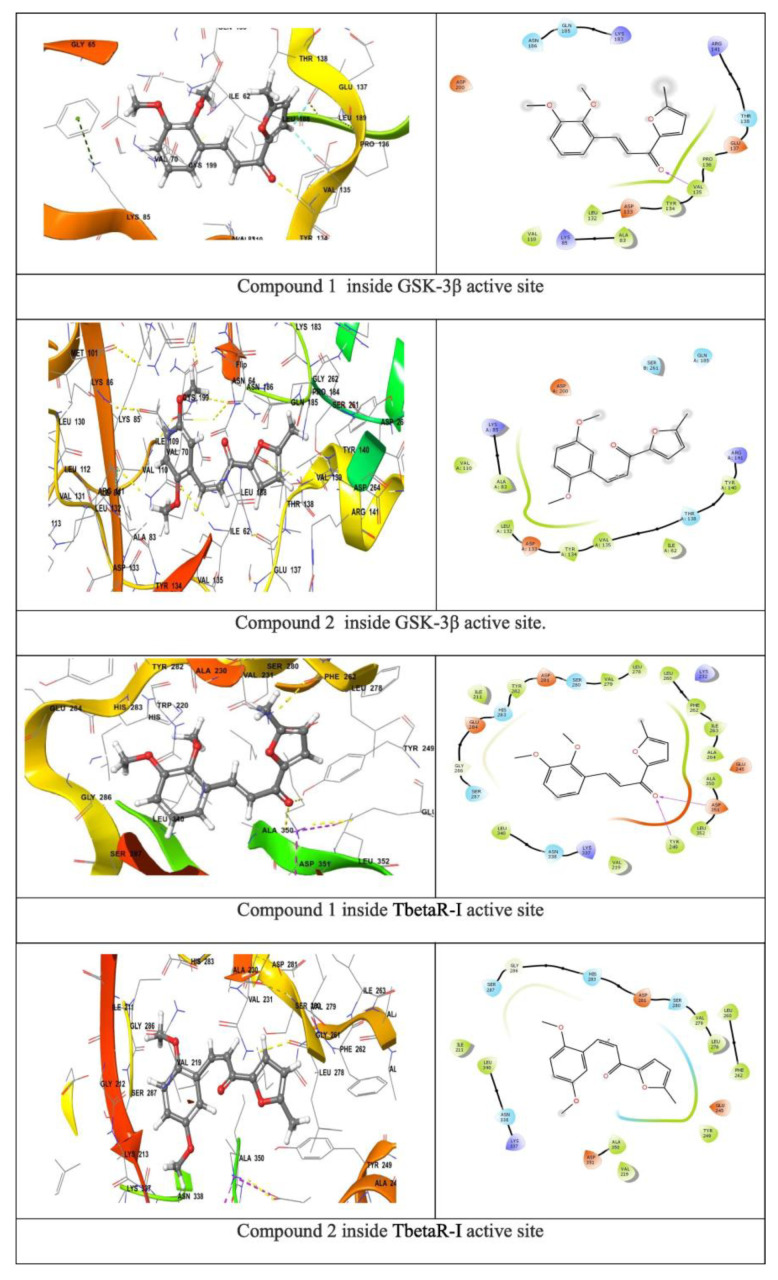
Different curcumin derivates depicting similar high binding affinity inside transforming growth factor beta type 1 kinase domain (TGF-β) and glycogen synthase kinase-3 beta (GSK3-β) active sites observing their role in wound healing (reprinted with permission [56] from Elsevier).

Researchers are developing new antibacterial phytoconstituents to combat drug-resistant bacteria. Researchers aim to synthesize curcumin derivative (CD) -nanofibers, evaluate its antimicrobial property by the disk diffusion method, and analyze its involvement in wound healing when interacting with TGF-β and GSK3-β. In silico studies (molecular docking and molecular dynamic) were employed to determine the most effective dual-action molecules on TGF-β and GSK3-β; the two most active compounds were produced and evaluated by 1H and 13C (NMR) and IR spectroscopy. By adding PVA as a polymer to ethanol electrospun CDs, the antibacterial effect of electrospun nanofibers against Staphylococcus aureus was investigated and revealed an elevated inhibition zone from 0.5 to 5.0 mg preloaded CD with the maximum inhibition zone of 8.5 ± 0.71 and 9.67 ± 0.29 mm for compound 1 (3-(2,3-dimethoxyphenyl)-1-(5-methylfuran-2-yl) prop-2-en-1-one) and compound 2 (3-(2,5-dimethoxyphenyl)-1-(5-methylfuran-2-yl)prop-2-en-1-one). When assessing the binding affinity of a ligand to a receptor, a scoring function has been used; a high negative value implies a strong binding energy between the compounds under study and the receptors. Based on their docking scores at the TbetaR-I protein’s binding region, compounds 1 and 2 have been found to have exceptional wound-healing activity. CDPVA could be a potential source for developing antibacterial diabetic wound dressings, as represented in Figure 4 [56].

It has been reported and proven that traditional cellulose-based cotton gauze hinders wound healing. Researchers utilized cellulose nanofibers (CNF) with phytoconstituents (curcumin) to make a wound-healing three-dimensional porous aerogel. Synthesized cellulose nanofibers from phyto-waste are biodegradable and biocompatible. A biopolymer (sodium alginate) was employed to retain mechanical strength. The morphology of nanofibers was assessed by SEM and assured macro- and microporous armature and phyto constituency and that curcumin improved wound healing. In vitro drug release studies have shown a sustained release pattern. The three dimensional (3D) nano-bio aerogel containing curcumin enhanced fibroblast migration and demonstrated antibacterial action against pathogens. Angiogenesis transpired in vivo without scaffold inflammation and, from the findings, it was deduced that this 3D porous aerogel could repair chronic wounds [57].

### 3.2. Aloe Vera-Incorporated Nanoplatforms

Since ancient times, aloe vera (AV) (Liliaceae family) has been used to treat a variety of skin ailments, including burns, infections, and other irritations. The leaf’s gel, which consists of 99% water, helps relieve dry skin. Since AV contains prostaglandin and bradykinin enzymes, inflammation is mitigated. At the same time, the glycoprotein component of AV has been demonstrated to promote the growth and migration of keratinocytes and fibroblast cells, making it an effective wound-healing agent. Research indicates that AV improves wound healing by increasing collagen content. The in vitro cytotoxicity utilizing the MTS assay and the wettability of an AV-loaded electrospun polycaprolactone (PCL) wound-dressing material was studied by Agnes et al., in 2015. Based on the results of the study, the material could be used as a wound dressing in situations when cell growth is necessary. The consistency of the material, as per the investigations, lasts for at least five days, which is thought to be the best standard for wound dressings [58]. The scientists next examined the efficacy of PCL mats and PCL/AV (15%) -integrated nanofiber scaffolds in promoting wound healing in both in vitro and in vivo models. This research suggested that the dressings were biologically compatible and might be used for this purpose. Following the in vivo wound-healing model, it was explicitly stated that increasing the AV concentration had no negative effect on hemolysis and that the percentage of hemolysis of the PCL- and AV-loaded membranes (5, 10, and 15%) were all found to be within normal limits. Histological examination of the in vivo experiment using nanofiber membranes infused with aloe vera (AV) extract revealed much faster healing in that group compared with the others. Following the 11th day of close observation, the wound began to heal and tissue formation was visible, moving at a faster rate than it had for the PCL and open wound. Healing was slower and inflammation and infection were more prevalent in the control groups than in those using dressings containing herbal extracts. From the literature, keratinocyte cells can easily migrate towards the outer wound layer to produce the scab and epidermal cells can move more rapidly in a moist wound than a dry wound. Nanofibers with AV extract provided a more hydrophilic environment to the wound, which accelerated the healing process. Researchers found that an AV extract-infused nanofiber wound dressing promoted a more hydrophilic environment. Additionally, nanofiber membranes improve cell adherence, nutrition delivery, and pathogen protection [59]. Silva et al. produced chitosan/AV membranes and investigated their degradation behavior and cells exposed as a potential healing. The extracted AV gel was added to the Cht solution at ratios of 2:1 and 1:1 *v*/*w* Cht/AV, termed CAV and CAV1, respectively. The authors proposed that incorporating AV gels into chitosan produced membranes with sufficient roughness, degradability, and mechanical characteristics. The findings from the bacterial cell suspension method (Figure 4) indicated that the antibacterial activities of CAV and CAV1 were better than that of Cht membranes, suggesting that the addition of AV to Cht enhanced the antimicrobial potential of the resulting membranes. However, statistical differences (*p* < 0.05) were not found between the CAV and CAV1 membranes. The biological activity of Cht/AV-based membranes can also be influenced by physical features such as the surface energy, topography, and stiffness, which directly influence protein adsorption and, subsequently, the cell response. In vitro biological investigations demonstrated that the membranes provided a favorable microenvironment for the adhesion, proliferation, and survivability of human skin fibroblasts [60]. Marziyeh Ranjbar Mohammadi et al. investigated the inclusion of AV into nanofibers manufactured from a biopolymer (gum tragacanth) and a synthetic polymer (PCL). The scientists claim that the PCL/ gum tragacanth (GT)/AV nanofibers possess adequate mechanical strength and hydrophilic nature, as well as a stable structure following biodegradation testing, which is a critical need for wound dressings. Scanning electron microscope results showed that increasing aloe vera to the nanofiber composition decreased the average diameter from 1118 ± 53 to 501 ± 69 nm for poly(ɛ-caprolactone) and from 184 ± 34 to 123 ± 22 nm for poly(ɛ-caprolactone)/gum tragacanth mats. Fourier transform infrared spectroscopy and differential scanning calorimetry analyses revealed that aloe vera was properly loaded in the nanofiber structure. With the addition of aloe vera, the tensile strength and tensile strain of poly(ɛ-caprolactone)/gum tragacanth nanofibers increased from 0.21 to 0.75 and from 25 to 32%, respectively. Aloe vera-loaded nanofibers also exhibited satisfactory degradation and wettability. Cell culture investigations with AV-loaded nanofibers revealed excellent attachment and proliferation. Because of its inexpensive cost, the hydrogel characteristics of GT, as well as the moisture absorption of scaffolds, the PCL/GT/AV material can also be utilized as a dressing for moderate to high exudate wounds [61]. Despite the fabrication of nanofibrous meshes comprising aloe vera, researchers have also fabricated varied systems as carriers for wound-healing applications, e.g., Suganya and colleagues proposed that AV-incorporated PCL has superior biological and healing characteristics when compared with a PCL/collagen combination for dermal replacements [62]. A moist wound bed will allow growth factors and numerous cell types, including epithelial cells, to migrate, facilitating wound edge contraction. To create and maintain this environment, appropriate dressings come into play. There are four basic principles involved in choosing an optimal dressing. If a wound proves to be dry or desiccated, it will need hydration. If a wound produces excessive exudates, the fluid needs to be absorbed. If a wound has necrotic tissue or evident debris, it will need debridement. Lastly, if a wound is infected, it needs to be treated with the appropriate antibacterial agent. There are also several other factors that are important when choosing a dressing, such as providing protection to the peri-wound skin, forming an effective bacterial barrier, conforming to wound shape, producing minimal pain during application and removal, being free of toxic or irritant extractables, not releasing particles or nonbiodegradable fibers into the wound, and maintaining the wound at an optimal temperature and pH. R. Pereira et al. used the solvent-casting process to prepare nanohydrogel films made with alginate and AV. Films with a high concentration of AV (25%) had enhanced water absorption. In order to measure the films’ transparency, the light transmission method was employed. In accordance with the findings, the transparency of the films improved with an increase in the amount of aloe vera used (AG: 1.28 ± 0.28; AGA5: 1.60 ± 0.16; AGA15: 1.90 ± 0.25; AGA25: 2.11 ± 0.09). The films with a high percentage of aloe vera present both improved water absorption and in vitro degradation. These results also suggest that alginate/aloe vera hydrogel films can be potentially explored as wound dressings for dry and exuding wounds [63]. Pandimadevi and group conducted studies that supported this observation. The AV-incorporated chitosan nanofilm was fabricated in this investigation utilizing solvent casting. A chitosan film comprising 15% *C. officinalis* extract and 25% AV extract had exceptionally good qualities and might be utilized as a wound-healing composite, based on a comparison of the antibacterial and mechanical capabilities of the AV-integrated chitosan and the chitosan incorporated with extract from *Calendula officinalis* petals. Chitosan AV films outperformed chitosan *C. officinalis* films because of their bacteriostatic action and decreased activity against Bacillus subtilis (Gram-positive) bacteria [64]. Biopolymeric nanofibrous composites loaded with inorganic materials have indeed been widely researched as tissue engineering scaffolds due to their superior structural and mechanical, as well as biological, properties. Electrospinning was employed to synthesize magnesium ferrite (Mg-ferrite) nanofibers. Mg-ferrite nanoparticles were developed using the reverse micelle approach and electrospun into magnetic nanofibers with polycaprolactone (PCL) and AV. Mg-ferrite/PCL/AV nanofibers were morphologically consistent and structurally and magnetically strong. Experiments employing cell viability assays and SEM demonstrated that magnetic nanofibers supported 3T3 cell viability. Researchers anticipate the unique composite nanofibrous membranes proposed in this research can imitate the physical function and structure of tissue ECM components and provide magnetic and soluble metal ion properties in scaffolds to facilitate cell adhesion and tissue regeneration [65]. In another study, a tetracycline hydrochloride (TCH)-loaded blended composition of poly(caprolactone)/gelatin/aloe vera nanofibers was fabricated with hybrid blended structures and was effectively manufactured and compared for wound healing. FTIR and water contact angle measurements revealed that mixed and hybrid nanofibers were hydrophilic. Morphological SEM investigations showed that fibers of both compositions were homogeneous. In vitro tests showed that the hybrid sample had improved antibacterial activity against (*E. coli*) and biocompatibility and revealed that AV and TCH increased wound-healing properties [66].

### 3.3. Centella asiatica Derived Systems

**Figure 5:**
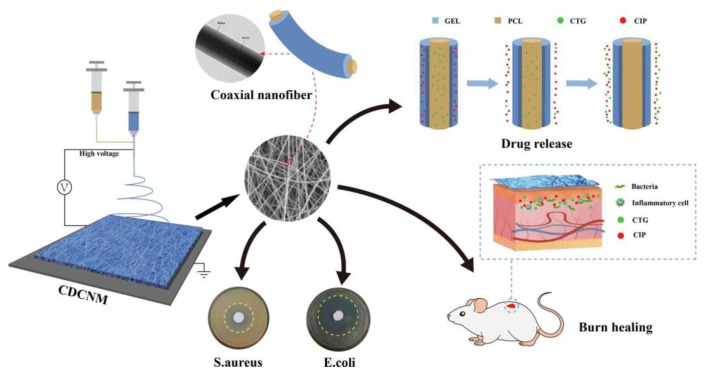
Representation of new centella total glucoside- and ciprofloxacin-based dual-loaded coaxial nanofiber membrane showing potent wound healing action (reprinted with permission [70] from Elsevier).

*Centella asiatica* (CA) is a medicinal plant commonly employed in traditional medicine to treat skin disorders and wounds, such as increasing wound healing, reducing keloids, preventing skin aging, and whitening skin [67]. The most well-studied activity of AS is wound healing, which is a complex process that involves three overlapping processes: inflammation, cell proliferation, and remodeling [68]. CA-loaded formulations are reported to be an efficient wound dressing. In one study, its extract was used as a reducing and stabilizing agent during the synthesis of CA-coated silver nanoparticles (CA-AgNPs). The composition of CA-AgNPs containing polycaprolactone (PCL) and polyethylene oxide (PEO) nanofibers was synthesized using the mutual electrospinning technique. A new wound dressing material was formulated using the mechanical qualities of PCL:PEO, the antibacterial action of AgNPs, and the wound-healing properties of CA. Additionally, the in vitro disintegration profile and silver release data indicate that these nanofibers could eventually be applied to wound dressings. CA-AgNPs appeared to be spherical and the average particle size was determined as 14.8 ± 7.3 nm. In an FTIR spectrum of CA extract, vibrations were observed at 1375 cm^−1^ and 1605 cm^−1^ due to C-O groups of carboxylic acids and phenols. The peak at 3288 cm^−1^ was attributed to the stretching of free O-H groups in phenols. The FTIR spectrum of CA-AgNPs was similar to the spectrum of CA, but, with the formation of CA-AgNPs, changes occurred in peak intensities at 1375, 1605, and 3300 cm^−1^. In the XRD diffractogram of CA-AgNPs-PEO/PCL nanofibers, at angles 2θ of 19.36° and 21.83° were the characteristic peaks of PEO and PCL, respectively. The increase in intensity observed at 23.70° was due to the mixture of PCL and PEO. The peaks at angles 2θ of 38.12°, 45.132°, 64.17°, and 75.97° belonged to crystalline AgNPs. The thermogram shows that the melting points of PCL and PEO nanofibers were 58.22 °C and 68.16 °C, respectively. For electrospun PCL/PEO nanofibers, double melting peaks belonging to PCL and PEO appeared at 56.93 °C and 65.92 °C, respectively. The PEO/PCL nanofibers offered a WVTR of 2545.71 ± 99.42 g/m^2^/day, whereas the nanofibers containing 10% CA-AgNPs presented a WVTR of 2202.12 ± 86.70 g/m^2^/day. Scholars reported that a daily WVTR of approximately 2000–2500 g/m^2^ would provide sufficient moisture balance in the wound area without causing dehydration. PEO/PCL nanofibers with 5% and 10% CA-AgNPs have been shown to be efficient against *Staphylococcus aureus*, *Escherichia coli*, and *Candida albicans* in antimicrobial investigations [69]. The coaxial electrostatic spinning method was employed for the synthesization of the *Centella asiatica* total glucoside–ciprofloxacin dual-loaded coaxial nanofiber membrane (CDCNM). Researchers have loaded centella total glucoside (CTG) and ciprofloxacin (CIP) into different fiber positions and evaluated the nanofiber membranes’ shape and coaxial structure by SEM and TEM to meet individualized therapeutic demands by altering release behaviors. Water contact angle, water absorption, breathability, and in vitro drug release were investigated. CDCNM promoted fibroblast growth in vitro and revealed good antibacterial activity by the agar flat dish diffusion method. CDCNM also enhanced rat-scald healing by boosting neovascularization and endothelial cell proliferation. Immunohistochemical staining demonstrated that CDCNM increased CD31 and VEGF expression during early wound healing. As a topical multifunctional wound dressing, this dual drug-loaded nanofiber membrane accomplished healing effect and continuous bacterial suppression, which presents innovative approaches for conventional trauma treatment tools and the dual drug delivery systems, as shown in Figure 5 [70].

**Figure 6:**
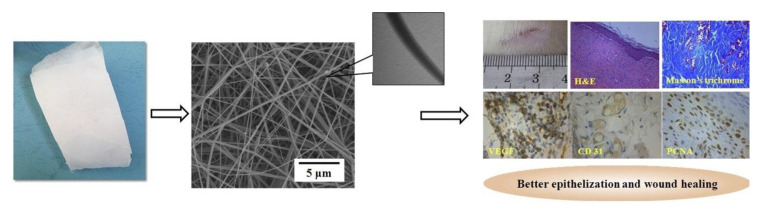
Illustration of asiaticoside-loaded coaxially electrospinning nanofibers containing alginate, polyvinyl alcohol (PVA), and chitosan in deep partial-thickness burn injury. (reprinted with permission [71] from Elsevier).

Asiaticoside-loaded alginate/PVA/chitosan coaxially electrospun nanofibers were synthesized by the coaxial electrospinning technique and further evaluated. SEM and TEM examined nanofiber morphologies and microstructures (TEM). Asiaticoside-loaded coaxial nanofibers were tested on deep partial-thickness burn injuries. Coaxial nanofibers were investigated for in vitro drug release. In vitro coaxial nanofibers released drugs faster, aiding wound healing. The alginate/PVA/chitosan coaxial nanofibers showed faster drug release rates and more drug release content than the centella triterpenes cream. Morphology, wound healing ratio, and pathological sections showed that they healed rats with deep partial-thickness burns. The prepared nanofibers possessed a core–shell structure, smooth surface, and uniform diameter. They healed rats with severe partial-thickness burns and treated profound partial-thickness burns, as shown in Figure 6 [71]. 

### 3.4. Azadirachta Indica-Incorporated Wound Dressings

Researchers have prepared *Azadirachta indica*-loaded nanofibrous mats as a wound dressing and further examined the antimicrobial and antibiofilm capabilities of an electrospun cellulose acetate nanofiber mat containing green-produced silver nanoparticles (CA-g-AgNPnanomat) and extract of *Azadirachta indica*. SEM, TEM, XRD, Fourier transform infrared spectroscopy, and contact angle measurement were used to characterize electrospun CA and CA-g-AgNPnanomat. The CA-g-AgNPnanomat’s antibacterial activity was assessed by a colony-forming unit (CFU) counting and disk diffusion assay. CA-g-AgNPnanomat antibiofilm capabilities were also examined. With improved NP internalization, the CA-g-AgNPnanomat was more effective against Gram-positive *Staphylococcus aureus*. MH-S macrophage cell lines of Musmusculus assessed the CA-g-AgNPnanomat’s biocompatibility. CA-g-AgNPnanomat decreased biofilm growth by 50%. CA-g-AgNPnanomat also reduced CFU/mL. The CA-g-AgNPnanomat, with strong biofilm activity, has great potential for healthcare, antimicrobial nanomat, and wound-dressing design [72]. The mat had 18.78 N tensile strength and 4.98 mm elongation. Neem extract in the nanofiber mat formed an inhibitory zone that synergistically inhibited bacterial cells. Thus, the novel nanofibrous mat may be ideal for wound dressing [73]. Neem gum polysaccharide (NGP) and polyvinyl alcohol (PVA) were blended to produce a nanofiber mat using the electrospinning technique. G14 (CL) was identified as the optimal grade after optimization was conducted based on the architecture (FE-SEM) and mechanical characteristics (tensile strength) of the fiber. The diameter range of fibers was measured using Image J software and the dimensions of the fiber were in the range of 100 to 300 nm for drug-free fibers and the diameters of the fiber shifted to the higher side (200–450 nm) on the incorporation of drugs. Antibacterial activity was tested against standard strains of *Pseudomonas aeruginosa* MTCC 2297, *Staphylococcus aureus* ATCC933, and Escherichia coli (IP-406006). The nanofibers were bactericidal to the testing microorganisms due to the strong antibacterial ability of bark extract from *T.undulata*. The results indicate that a herbal drug-loaded PCL/PVP nanofiber mat possesses efficient antibacterial property and can be used in the treatment of wound healing or dermal bacterial infections, thereby proving a potential application for use as drug delivery and as a wound dressing agent. The produced nanofiber mat (NFM) was discovered to be hemocompatible and biodegradable and to have antibacterial action against *S. aureus* and *E. coli*. Thermogravimetric analysis (TGA) curves were used to analyze the thermal degradation of fibers made from a PCL/PVP blend and PCL/PVP loaded with herbal drugs. After heating from 40 °C to 800 °C at a rate of 20 °C per min under a nitrogen purge 200 mL per min, a total of 1.5 mg of PCL/PVP blend and herbal drug-loaded PCL/PVP mat was extracted. In contrast to the PCL/PVP blend without the herbal drug, which only results in a first weight loss from 391 to 431 °C, the PCL/PVP degradation with the herbal drug results in a weight loss at two distinct temperatures initially, from 212 °C to 281 °C, followed by a second loss from 385 °C to 413 °C. The optimal grade was described using FTIR, TGA, and OCA, among other analytical methods. The effectiveness of nanofiber mat (NFM) air filtering was also studied. For skin tissue engineering, the NFM demonstrated quicker healing for wounds [74].

### 3.5. Tecomella undulata Extract-Based Systems for Enhanced Wound Healing

Antibacterial or wound-healing medicinal chemicals can be carried by nanofibrous membranes, which are soft and have high surface-to-volume ratios. A PCL/PVP nanofiber mat comprising a chloroform: methanol (4:1) crude bark extract of *Tecomella undulata*, a bioactive compound used for wound healing, was synthesized and tested for antibacterial activities. As seen under the microscope, neither the drug-free nor the drug-loaded nanofibers were affected by the incorporation of herbal extract into the polymeric matrix, suggesting that the form of the resulting fibers was unaffected. Standard *Pseudomonas aeruginosa* (MTCC 2297), *Staphylococcus aureus* (ATCC 933), and *Escherichia coli* strains were used to determine the level of effectiveness (IP-406006). It was found that a PCL/PVP nanofiber mat loaded with extract may suppress bacterial growth, making it a promising candidate for use as a wound dressing and drug carrier [75]. Antibacterial, moisture vapor transport, in vitro drug release, and wound healing properties of a PCL nanofibrous membrane containing the herbal drugs *Tecomella undulata* (TU), *Glycyrrhiza glabra* (GG), *Asparagus recemosus* (AR), and *Linum usitatissimum* (LU) were evaluated. Incorporating herbal drugs into a PCL polymer matrix had no macroscopic effect on either the drug-free or the drug-incorporated nanomembrane. The herbal medication-incorporated PCL nanomembrane inhibited bacteria growth, indicating that it might be used as a wound dressing and as a drug delivery method [76].

**Table 1:** Various herbal bioactive-loaded nanofibrous meshes with their properties.

Phytoconstituent	Biopolymer	Formulation	Method of Fabrication	Properties of the Prepared Formulation	References
Curcumin	Gelatin	Indomethacin/gelatin/curcumin nanofibers	Electrospinning	Antioxidant, anti-inflammatory, analgesic properties	[77]
	Collagen	Curumin-loaded chitosan/poly(ethylene oxide)/collagen (Cho/PEO/Col) nanofibers	Blend–electrospinning process	Anti-inflammatory and anti-infective properties	[78]
	Chitosan	Chitosan/curcumin@β-cyclodextrin/silver nanoparticles (CS/Cur@β-CD/AgNPs)	Electrospinning	Antibacterial and antiscarring properties.	[79]
	Alginate	Alginate/gelatin sponge combined with curcumin-loaded electrospun fibers (CFAGS)	Electrospinning and interpenetrating polymer network (IPN) strategy	Antibacterial properties	[80]
	Cellulose	Graphene oxide/TiO_2_/curcumin-incorporated cellulose acetate (CA) nanofiber	Electrospinning	Biocompatibility and antimicrobial	[81]
	Hyaluronic acid	Bioinspired hyaluronic acid blends immobilized with 3, 4-difluorobenzylidene curcumin (CDF) non-woven nanofiber mats	Electrospinning	Antimicrobial, antibacterial, and anticancer properties	[82]
Aloevera	Gelatin	Hybrid nanofibers fabricated from gelatin/aloevera/poly(ε-caprolactone	Double nozzle electrospinning	Antibacterial activity	[83]
	Collagen	Zein/Polycaprolactone/Collagen nanofibers	Electrospinning	Anti-infective properties	[84]
	Chitosan	Polycaprolactone/chitosan/aloe vera nanofiber membranes	Sloping free surface electrospinning	Antibacterial activity	[85]
	Alginate	Aloevera and aqueous leaf extracts of *Moringa oleifera* calcium alginate-PEG-methyl ether methacrylate (PEGMA) scaffolds	Electrospinning	Anti-inflammatory properties and antimicrobial activity	[86]
	Cellulose	Tetracycline hydrochloride (TCH) -loaded poly(caprolactone)/gelatin/aloe vera nanofibers	Electrospinning	Antibacterial activity	[66]
	Hyaluronic acid	Ethylcellulose/hydroxypropyl methylcellulosenanofiber mats	Electrospinning	Antibacterial properties	[87]
Centella asiatica	Gelatin	Gelatin/chitosan/nonwoven fabric composite hydrogel wound dressing		Antibacterial activity	[88]
	Collagen	*Centella asiatica*/silver nanoparticle (CA-AgNPs) -loaded Poly caprolactone/ polyethylene oxidePCL/PEO hybrid nanofibers	Mutual electrospinning method	Antimicrobial activity	[69]
	Chitosan	Double-layered Polycaprolactone/Poly(vinyl alcohol)_Chitosan-Sodium tripolyphosphate_*Centella asiatica* (PCL/PVA_CS-TPP_CA) bionanocomposite dressing material	Electrospinning	Anti-inflammatory and antibacterial properties	[89]
	Gelatin/collagen/cellulose	Asiatic acid	Electrospinning	Antimicrobial	[90]
	Cellulose	Cellulose acetate/centella asiaticananofibers	Electrospinning	Antibacterial activity	[91]

## 4. Natural Polymer-Based Nanofibrous Matrices

Biodegradability, biocompatibility, and low antigenicity are the properties of natural polymers. Some have antibacterial and anti-inflammatory properties and can aid tissue repair; this boosts the interaction of nanofibers with natural substances involved in the healing process, making them a popular choice for electrospinning wound dressings [92]. Natural polymers have worse mechanical properties and are more expensive and difficult to process than their synthetic counterparts, making them less desirable for use in electrospinning. As a consequence, synthetic and natural polymers are synthesized in blended solutions or co-electrospun from different solutions to adjust the physical and chemical properties for highly specific wound-healing dressings. Natural polymers such as gelatin, collagen, chitosan, alginate, cellulose, and hyaluronic acid play an important role in wound healing [93,94,95].

### 4.1. Gelatin

Gelatin is a natural protein biopolymer made by hydrolyzing a portion of collagen; it appears as translucent yellow particles or flakes [96]. Products can be dissolved in boiling water, glycerin, and acetic acid and they are numerous and inexpensive. Biocompatible and biodegradable, it is often used in tandem with the synthetic polymer polycaprolactone (PCL) to boost performance [97]. At a physiological pH, Type A gelatin is positively charged after acidic hydrolysis, while Type B gelatin is negatively charged after alkaline hydrolysis. Due to its widespread application as a drug delivery system for growth factors, crosslinking gelatin has been explored for its capacity to modify the degradation and release rate of encapsulated dosage forms [98]. In 2018, Rather and coworkers used electrospinning to develop a mesh of cerium oxide nanoparticle (CeNP) -functionalized polycaprolactone (PCL) gelatin nanofiber (PGNPNF). The incorporation of CeNPs into the PGNPNF mesh resulted in a SOD-mimetic activity. When tested with Alamar blue, the PGNPNF mesh increased 3T3-L1 cell growth by 48%. In conclusion, this study provides promising evidence for the use of CeNP-functionalized PCL-gelatin nanofibrous mesh in wound healing [99]. The antibacterial wound dressing developed by Lin and colleagues in 2022 is a coaxial polycaprolactone/gelatin (PCL/GEL) nanofiber mat containing ciprofloxacin loaded into the PCL (core layer) and tetracycline hydrochloride loaded into the GEL (shell layer). The results showed that the ciprofloxacin has a slow release over five days (80.71%), while the tetracycline hydrochloride had a fast release over 12 h (83.51%). The coaxial nanofiber mesh was found to have significant antibacterial action against *E. coli* and *S. aureus* in in vitro experiments. Moreover, the coaxial mats demonstrated enhanced biocompatibility toward human skin fibroblast cells (hSFCs) [100].

### 4.2. Collagen

Collagen is the most common protein in mammals and the major molecule responsible for giving the extracellular matrix its structural integrity. It is also biocompatible and has a low antigenicity. Collagen’s pro-hemostatic and proliferative properties in fibroblasts and keratinocytes have made it a popular component in skin substitute materials and wound dressings. Many other fluoroalcohol-based solvents, such as 1,1,1,3,3,3-hexafluoro-2-propanol (HFP) and 2,2,2-trifluoroethanol (TFE), as well as ethanol-in-water solutions, have been used successfully in the electrospinning process. The poor mechanical qualities and high breakdown rates after implantation of collagen nanofibers have been improved by co-electrospinning with various synthetic and natural polymers [98]. Khandaker et al., 2022, developed a polycaprolactone nanofiber mesh by an electrospun method and examined its efficacy as a cutaneous drug delivery route in mice. Both glutathione (GSH)-immobilized PCL meshes showed outstanding biocompatibility and exhibited no degradation through day 20 of the study period in fibroblast cell adhesion and cytotoxicity assays. Experimental evidence showed that GSH was released more slowly from a PCL-GSH-GLU complex in comparison with a PCL-GSH-PBS mesh after being attached to PCL via a glutathione–glutaraldehyde (GSH-GLU) complex. This study confirmed the benefit of employing PCL mixed with GSH and GLU as a transdermal anti-oxidative and antibacterial medication delivery system for bandages, skin grafts, and other wound healing applications in diabetic patients [101]. The group led by Xiang created an electrospun membrane that resembled a mesh by employing a copper mesh receiver. Water contact angles for the mesh-like scaffolds with and without atorvastatin were 126.81° ± 2.65 and 132.27° ± 0.75, respectively, whereas for the random fibers (PCL/R) it was 129.29° ± 0.60. On day one, the atorvastatin release rate from the polycaprolactone mesh-like membrane (PCL/MAT) was ~ 43.69 ± 2.13%. After four days, >65.47 ± 1.96% of the atorvastatin had been released from the membrane. In the subsequent 7 days, the atorvastatin was gradually and consistently released by the fibers. The drug-loaded fiber membrane platform release rate was 89.34 ± 2.16%. A healing ratio of PCL/MAT of 60.44 ±1.40% was found in in vivo animal studies [102].

### 4.3. Chitosan

Polysaccharide chitosan is created when chitin from crustacean shells is partially deacetylated. Chitosan refers to a class of chitosan polymers having deacetylation levels between 40% and 98% and molecular weights between 50 and 2000 kDa. Only organic acids (such as hydrochloric acid or glutamic acid) may dissolve chitosan, resulting in chitosan chloride salts and chitosan glutamate salts. As a result of protonation, chitosan’s amine groups acquire a net positive charge in solution. Because of its biocompatibility, low toxicity, and ability to be destroyed by naturally occurring enzymes such as chitosanase and lysozyme, chitosan has been widely used in a variety of biomedical applications [103,104]. In 2022, Hu et al. synthesized hydroxypropyl chitosan azide (AZ-HPCTS) and prepared it as a hydrogel coating applied to a polypropylene mesh (PPM) using UV irradiation. The results of this investigation corroborated previous research indicating that hydrogels with porous three-dimensional network architectures are biocompatible, biodegradable, and have strong adhesion to PPM [105]. In 2022, Liu and his co-researchers developed a composite chitosan electrospun nanofibrous material that included curcumin, β-cyclodextrin, and silver nanoparticles (Cur@-CD/AgNPs). Curcumin was released from Cur@-CD/AgNPs in vitro at a cumulative rate of 52.8% in 5 h when dialyzed in a phosphate buffer (pH 5.5) and at a rate of 72.4% in 48 h when dialyzed in a buffer with a pH of 7.4. Nanofibers loaded with Cur@-CD/AgNPs and lysozyme were shown to be highly biodegradable for use as a wound dressing, as determined by a mass loss testing performed in a physiological solution with and without lysozyme at 37 °C for 14 days. When compared with PBS, the hemolysis rate of 2 mg/mL nanofiber was determined to be lower than 3%. After 24 h, almost 80% of the L929 cells that were co-incubated with silver nanoparticles and nanocomposites of different concentrations were still active [79]. Hydrophilic polyethylene oxide, chitosan/polyethylene oxide (PEO), and kaolin nanofiber membranes were developed by Liu et al., in 2022, using the electrospinning technique (CPKs). Results from in vitro coagulation experiments demonstrated that CPKs with a 10% kaolin ratio (CPK10) had exceptional hemostatic efficacy. Using a whole blood coagulation time (WBCT) assay, it was determined that the hemostatic time of CPK10 (43 ±1.4 s) was much lower than those of chitosan/polyethylene oxide (CPK0) nanofiber membranes (61 ± 2.2 s) and QuikClot^®^ combat gauze (55.7 ± 1.2 s). Further testing on rat liver damage indicated that CPK10 can control bleeding more quickly and effectively than other groups. Furthermore, CPKs accelerated the recovery of rats with a back wound in just 14 days with minimal inflammation [106].

### 4.4. Alginate

Brown seaweed is the source of alginate, an anionic polysaccharide. As a whole, it consists of (1,4)-linked-D-mannuronate (M) and -L-guluronate (G) residues. So, alginate is a linear unbranched copolymer with blocks of consecutive G residues, blocks of consecutive M residues, and blocks of alternating M and G residues [107]. The molecular weight and physical properties of alginate, which range from 50 to 100,000 kDa, are determined by the ratio and sequence of these residues, which in turn depend on the source of the alginate. Alginate is superior to other options since it is safe, inexpensive, and biocompatible and has hemostatic and absorbent qualities. Because of these features, it has found widespread use in biomedical applications such as drug delivery and tissue engineering [108,109]. Using electrospinning technology, Lu and his team in 2021 developed a natural antimicrobial nano composite fibers of alginate and oregano essential oil. The diameter of the prepared fibers varied from 38 mm to 105 mm. Alginate/oregano composite nanofibers were subjected to an extensive antimicrobial study using a plethora of Gram-positive (methicillin-resistant Staphylococcus aureus (MRSA) and Listeria monocytogenes) and Gram-negative (Klebsiella pneumoniae and Salmonella enterica) bacteria, which are prevalent wound and food pathogens. Results showed that when the oregano essential oil content was raised from 2% to 3% wt, antimicrobial activity increased against all pathogens, with the highest growth rate seen in activity against MRSA when compared with a non-alginate-based control disk containing oregano essential oil [110]. Using 3D printing technology, researchers in another study developed mesh implants composed of alginate and waterborne-polyurethane (A-WBPU). To achieve this goal, five distinct formulations of aqueous polyurethane ink with varying quantities of alginate were prepared and their rheological properties were studied. Good printability is demonstrated across all ink formulations used in 3D printing, allowing for the production of surgical mesh implants with a wide range of morphological properties that may be tailored to each individual patient’s injury using computer-aided design (CAD). After 3D printing, a coating of calcium chloride (CaCl2) was applied to strengthen the mesh. A physiological tensile strength value of 16 N cm^−1^, showing sufficient elasticity to cover physiological corporal movements (42.57%), was determined by mechanical analysis to favor CaCl_2_-coated meshes containing 6 wt% of alginate in their formulation for utilization as implants for minor and pelvic hernias. It was observed that the developed A2.5-WBPU + CaCl_2_ with a 6 wt% of alginate in the printed mesh was the most suitable mesh for hernia repair application by comparing the values obtained for the 3D printed meshes with the reported physiological values of 16 N cm^−1^ for tensile strength and 20–40% for elastic elongation. Elastic elongation values of 42.57 ± 6.60% allowed the mesh to return to its former shape following physiological movements; the elastic limit was 15.83 ± 1.04 N cm^−1^, which is sufficient for supporting the physiological tensions of small and inguinal hernias. Cell survival rates more than 90% in the first week after being implanted into the selected A2.5-WBPU + CaCl_2_ mesh were observed, suggesting that this mesh could facilitate tissue healing of the injured area. Antibiotic-loaded meshes were fabricated with 3D printing technology and then administered locally in an effort to reduce the prevalence of surgical site infections. It was found through in vitro drug delivery assays using chloramphenicol-loaded A2.5-WBPU + CaCl_2_ that 80% of the antibiotic was released within the first 24 h post-implantation [111].

### 4.5. Cellulose

Cellulose is a biopolymer composed of β-D-glucose linked together by β-1, 4-glycosidic linkages; it is the most abundant biopolymer in plant cell walls. Because of its great flexibility, excellent physical barrier for microbiological pathogens, and especially its moisture-retaining properties due to the presence of many hydroxyl functional groups, cellulose has been widely employed in wound dressing products. After being wet spun, chitosan–cellulose aerogel microfibers (CHCLAFs) have been dried using supercritical carbon dioxide (scCO2) to be used as wound dressings in two different ratios, 1:5 and 1:10, respectively. Ibuprofen (IBU) was also infused into the fibers via a post-treatment scCO2 impregnation. The fibers were woven into meshes with a high absorption rate for water (~400 wt%) and bactericidal properties against Escherichia coli and Staphylococcus aureus. The hybrid fibers were able to release IBU in a sustained way over 48 h and there was no cytotoxicity when employing fibroblast cells to create the fibrous structures [112]. In 2022, Panthi’s group adopted chemical treatment methods to synthesize cellulose microfiber (CMF) from raw fiber. Bioactive plant extracts from *Catharanthus roseus* roots were encapsulated in a crosslinked cellulose fiber matrix (*C. roseus*). The XRD pattern revealed that the average crystalline size of the isolated cellulose microfiber was 2.53 nm, with a crystalline index of 30.4%, while the crystalline size and crystalline index of the membrane that included the plant extract were 2.49 nm and 27.99%, respectively. An increase of up to 250% in the membrane’s water-uptake efficiency was achieved during the synthesis process. The composite (the CMF-E membrane) was tested for its antimicrobial efficacy against Gram-positive and Gram-negative bacteria using the zone inhibition method; the results showed a high level of antibacterial activity [113].

### 4.6. Hyaluronic Acid

Hyaluronic acid (HA) is a polysaccharide composed of N-acetylglucosamine and D-glucuronic acid disaccharide units. As a major part of the extracellular matrix, it can be found in a variety of different tissues, including connective, epithelial, and neural ones [114]. In 1934, HA was found in the eye’s vitreous humor; in 1964, it was produced in a laboratory. In terms of molecular weight, HA encompasses a wide range, from around 2 × 105 to about 107 Da. The physical and chemical characteristics of HA can be affected by its average molecular weight. Because of its high concentration of hydroxyl groups, HA is a hydrophilic polysaccharide that readily absorbs water [115]. Hydrogen bonds allow it to attach to water molecules. Chemical alteration of HA is possible due to the presence of functional groups such as carboxyl, hydroxyl, and acetamido [116]. HA has a wide range of biological effects, including cell differentiation, embryological development, inflammation, wound healing, viscoelasticity, etc. [117]. In 2022, researchers developed nanostructured hybrid hydrogels to be used as wound dressings by combining well-defined maleoyl-chitosan/poly(aspartic acid) (MAC5/PAS) nanogels into a polymer network based on thiolated hyaluronic acid (HASH). Hydrogels were prepared using MAC5/PAS nanogels, which also served as nanocarriers for encapsulating amoxicillin and regulating its release via the HASH hydrogel network. The in vitro release profile showed that the amoxicillin release rate was highest at a pH of 5.4, suggesting that the pH of the surrounding environment can affect the drug’s efficacy. Hydrogels have been shown to be biocompatible in both in vitro and in vivo studies, demonstrating their potential as therapeutic scaffolds for use in dressing applications [118]. Thiolated hyaluronic acid (THA) conjugates were utilized to optimize a dual-network hydrogel containing silk fibroin and bioactive glass nanoparticles. In vitro cell culture testing revealed a significantly boosted migration of fibroblasts and human umbilical vein endothelial cells. Full-thickness skin defect (in vivo) showed that prepared hydrogel entirely replaced the skin defects with vascularized tissues and complete appendages in two weeks [119].

## 5. Patent Overview

There are various patented technologies based on phytoconstituent-related biopolymeric fibrous meshes as wound dressings that have been recently filed or granted (Table 2). In the most recent advancement, the inventers from Shandong University have explored the herbal extract of *Humifuse euphoria* along with sodium alginate and have formulated them into a fibrous biocomposite dressing. The product was found to be ecofriendly, non-toxic, and biocompatible and showed better mechanistic strength and helped in alleviating the burning medical waste problem [120]. Rongmei and group from Zhejiang Sci Tech University developed a process utilizing shaddock peel for pectin and oxidized chitosan into the fibrous composite hydrogel. The formulation exhibited better cell proliferative activity and provided a nutrient source along with functioning waste exchange for a smooth healing mechanism [121]. Interestingly, in 2022, a patent was published on a fibrous wood pulp-treated chitosan/alginic acid-based reliable cleaning material. This sandwich construct observed good strength and water retention effect [122]. Another group discovered a facile method of preparation for collagen-based lyophilized fibers using recombinant human type III. This fibrous formulation resulted in high water retention in skin membrane and enhanced tissue formation [123]. In a concomitant study, the researchers from Wuhan Nuowei Biotechnology Co Ltd. (Wuhan, China) explored polyester fibers as a non-woven gauze along with sodium alginate as a hemostatic agent that is adhered onto the surface. This non-woven hemostatic fibrous gauze showed a stable effect and improved healing action [124]. Recently, an application of hyaluronic acid in the presence of a self-assembled short peptide into a nanofibrous network structure for a wound-repair dressing in an ultraviolet rays’ skin injury has been filed by Chengdu Saienbei Academy of External Sciences, China [125]. In 2021, the coaxial electrospinning method was fabricated to encapsulate a chitin- and lignin-based fibrous gel with polycaprolactone. This can be further loaded with a therapeutic agent that helps in promoting the healing rate and controlled drug release, especially in case of severe wounds [126]. Another group of inventers worked on the physicochemical treatment of herbal extracts of *Curcuma longa*, *Emblica officinalis*, and *Camellia sinensis* nano formulated in the form of fibers/gel without using polymer, exhibiting multiple-use bandages in wound-healing applications [127]. Earlier, chitosan has been explored widely in order to construct sandwich nanolayer-based fibrous sheets using this biopolymer and herbal extracts such as *Melilotus officinalis* for wound-healing dressings [128]. A wound dressing that responds to stimuli consists of a lyophilized polymeric (hyaluronic acid) hydrogel with several devices embedded in it. Chitosan and hypromellose can be used to make biofilms and electrospun fiber mats, respectively, with each of these devices [129], as shown in Table 2.

## 6. Conclusions and Future Perspective

Consequently, the current study focuses on innovative methods of encapsulating phytoconstituents in polymeric scaffolds, which could be employed as a replacement for synthetic analogues in wound healing. The review article concentrates on the transformation of herbal products into acceptable formulations based on the use of herbal constituents. The review was created to present unique techniques of using phytoconstituents and applications of electrospinning; other nanofiber production processes must be investigated in order to comprehend the many ramifications. Our group believes that phytoconstituents should be investigated for their potential, which may be proved using a nanofiber composition. The nanofibers have been detailed in depth as a method for delivering herbal drugs/raw extracts for diverse purposes, as well as compiled for simple evaluation by researchers regarding the advancements in this domain. Unfortunately, due to a lack of scientific justification and processing challenges, such as extraction, standardization, and the identification of specific medicinal components in complex polyherbal systems, herbs have long been ignored in the creation of innovative formulations. When using herbal medicines, there are a number of obstacles that must be overcome, including the creation of straightforward bioassays for standardization, pharmacological and toxicological evaluation techniques, research into the sites of absorption, the use of toxic herbal drugs, and the identification of various animal models for toxicity and safety evaluation. Modern phytopharmaceutical research, on the other hand, may address pressing scientific issues such as figuring out which phytoconstituents are present and how to use herbal remedies to treat wounds. The need is to develop innovative ways for extracting active constituents from medicinal plants that have been clinically shown to be effective. This would facilitate the rapid integration of the developed product into the market by various federal agencies. The fundamentals of the nanofiber synthesis process must be grasped before developing a specific application.

## Figures and Tables

**Table 2:** Recent Patent Overview.

Sr. No.	Patent Details	Biopolymer Used	Goal	Reference
1.	CN114904041A (2022)	Sodium alginate	Biodegradable fiber-based medical dressing formulated, incorporating sodium alginate with *Humifuse euphoria* extract	[120]
2.	CN114712553A (2022)	Pectin and chitosan	Biopolymer-based composite hydrogel has resulted in excellent wound-healing rate, especially in terms of it surface area	[121]
3.	WO2022078081A1 (2022)	Chitosan and alginic acid	Treatment of wood pulp fiber with biopolymers led to the development of composite wear-resistant material	[122]
4.	CN114632022A (2022)	Collagen	Recombinant human type III collagen-related freeze-dried fiber showed increased water retention and improved tissue construct	[123]
5.	CN114748670A China (2022)	Sodium alginate	Polyester fibers (non-woven gauze) and sodium alginate (hemostatic material) are adhered on the substrate exhibiting stable action and better wound-healing effect	[124]
6.	CN114989249A (2022)	Hyaluronic acid	The short peptide was self-assembled into nanofibers for wound repair dressing in ultraviolet rays’ skin injury	[125]
7.	US11124897B1 (2021)	Chitin and lignin	Polycaprolactone-coated biopolymer-based nanofiber scaffolds loaded with antimicrobial agent promoted chronic wound-healing property	[126]
8.	ES2885052T3 (2021)	*Curcuma longa*, *Emblicaofficinalis*, and *Camellia sinensis* extracts	Physicochemical treatment of natural compounds in the form of fibers/gel without using polymer, observing multiple-use bandage for wound-healing application	[127]
9.	US9101508B2 (2015)	Chitosan	Sandwich nanolayers have been constructed into fibrous sheets using biopolymer with *Melilotusofficinalis* for wound dressing	[128]
10.	US10080816B2 (2018)	Lyophilized hyaluronic acid (HA) hydrogel	Stimuli-responsive wound dressing containing a lyophilized polymeric (hyaluronic acid) hydrogel and several devices inserted in it, each gadget can create biofilms or electrospun fiber mats from chitosan and hypromellose	[129]

## Data Availability

Data sharing not applicable. No new data were created or analyzed in this study. Data sharing is not applicable to this article.
